# The cost of medical conferences

**DOI:** 10.3332/ecancer.2012.ed14

**Published:** 2012-05-18

**Authors:** Linda Cairns

**Affiliations:** Science Editor, ecancermedicalscience, European Institute of Oncology, Milan, Italy

Dr John Ioannidis has spent his career challenging his peers by exposing their bad science and questioning everything from surgical habits to drug studies. He has now decided to attack a major cultural institution of medicine: the conference. In a provocative piece in JAMA (the Journal of the American Medical Association) [1] he questions the value of attending scientific conferences. It’s a theme that re-emerges often during times of tight budgets. He estimates that there are probably more than 100,000 medical conferences a year if you include local meetings and he asks:

*“Do medical conferences serve any purpose? In theory, these meetings aim to disseminate and advance research, train, educate, and set evidence-based policy. Although these are worthy goals, there is virtually no evidence supporting the utility of most conferences. Conversely, some accumulating evidence suggests that medical congresses may serve a specific system of questionable values that may be harmful to medicine and health care”*.

One of the points he raises is travel. Ioannidis cites a 2008 commentary in the BMJ [2] that estimates the jet-fuel pollution at around 10,000 tons of carbon for the attendees’ roundtrip flights to a mid-sized international conference.

So one obvious alternative is video-conferenced meetings. Ioannidis continues:

*“In the electronic age in which information can be shared around the world instantly, the contribution of large medical conferences to the dissemination and advancement of science is unclear. Education and training can also happen outside of such venues. A portion of the resources spent on congresses and their accompanying extravaganzas could be better spent developing more efficient educational modes”*.

*“Eventually, some evidence should be accrued on whether specific types of current conferences offer advantages compared with other means of serving the same needs, including social networking tools, remote conferencing, and re-purposed meetings”*.
